# Supplementary material for: A role for the ATP-dependent DNA ligase lig E of Neisseria gonorrhoeae in biofilm formation
Source: BMC Microbiol. 2024 Jan 20;24:29. doi: 10.1186/s12866-024-03193-9 (PMC10799422; doi:10.1186/s12866-024-03193-9)
Supplement: Supplementary file 1 — Supplementary Material 1 [file 12866_2024_3193_MOESM1_ESM.pdf]

A role for the ATP-dependent DNA ligase Lig E of *Neisseria gonorrhoeae* in biofilm formation

Jolyn Pan[1], Avi Singh[1], Kyrin Hanning[1], Joanna Hicks[2], and Adele Williamson[1] *

[1] School of Science, University of Waikato, Hamilton, New Zealand
[2] School of Health, University of Waikato, Hamilton, New Zealand

* To whom correspondence should be addressed:
Adele Williamson
adele.williamson@waikato.ac.nz
ORCID 0000-0001-8139-1071

SUPPLEMENTARY INFORMATION

Supplement 1. Primers for mutant confirmation and sequencing

Primer name	Sequence (5' to 3')	Purpose
Primer_1_KO_FD	CGGGGAGAATTTCGTAACGT	Lig E forward primer
Primer_2_Rev	CACTTTTTGCAGTGCGGCA	Lig E reverse primer
Primer_3_GFP_FD	AACTTTTCACTGGAGTTGTCCC A	Lig E-GFP forward primer
Primer_4_His_FD	CACCACCACCACCACCACT	Lig E-His forward primer
Primer_seq_Lig-E_Fd	CAAATCAGGGTGCAGTTTTGGC GGAA	Lig E forward sequencing primer
Primer_seq_Lig-E_Rev	GCCCCGCCCATCACGGGCAGG AGCA	Lig E reverse sequencing primer
NS10_Construct_Fd	GTCCGCAATGCGCCGAAT	NS10 forward primer
NS10_Construct_Rev	GCCTGTCCGCGTCTGAAA	NS10 reverse primer
NS10_External_Fd	GTAACGGTTTTCAATGCC	NS10 forward sequencing primer
NS10_External_Rev	GATATGCGCGGACATTAT	NS10 reverse sequencing primer

Supplement 2. qPCR primers and probes

Oligonucleotide name	Sequence (5' to 3')	Purpose
Lig E primer Fd	CGTATTGGGACGGAAAGCA	Lig E gene forward primer
Lig E primer Rev	AATCTGCTCGAACTGACCA C	Lig E gene reverse primer
Lig E probe	CAAAGGCTTTACCGCGCAG TTTCC	Lig E gene probe
16s primerFd	CTGGGATAACACTGACGTT CAT	16s gene forward primer

16s primer Rev	GCAATCAAGTTGCCCAACA G	16s gene reverse primer
16s probe	AGTCCACGCCCTAAACGAT GTCAA	16s gene probe

Supplement 3. Mauve alignment of knock-out and Wild-type highlighting disruption of Lig E

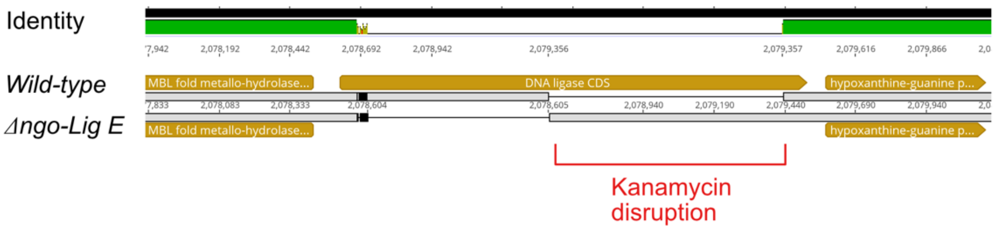

Supplement 4. Oligonucleotide sequences for generating ligatable double-stranded DNA substrates. Combinations used for different ligatable breaks are given in Supplement 5

**Oligonucleotide**	**Composition**
L1	5’-(6-carboxyfluorescein) AGGCCATGGCTGATATCGCA-3’
L2	5’-(phosphate) TAGGCATTCGAGCTCCGTCG-3’
L3	5’- CGACGGAGCTCGAATGCCTATGCGATATCGGCCATGGCC T -3’
L6	5’-CGACGGAGCTCGAATGCCTA-3’
L7	5’-(phosphate) TGCGATATCAGCCATGGCCT-3’
L8	5’-(phosphate) ATATCAGCCATGGCCT-3’
L9	5’-CGACGGAGCTCGAATGCCTATGCG-3’
L10	5’- CGACGGAGCTCGAATGCCTACGCGATATCAGCCATGGCC T-3’
L11	5’- CGACGGAGCTCGAATGCCTAGTGCGATATCAGCCATGGC CT-3’

Supplement 5. Oligonucleotide combinations used to generate different double-stranded ligatable substrates. Sequences are given in Supplement 4.

**Substrates**	**Ligatable strand**	**Complementary strand**
Single nick	L1, L2	L3

Blunt ended	L1, L2	L6, L7
Overhang	L1, L2	L8, L9
Mismatch	L1, L2	L10
Gapped	L1, L2	L11

Supplement 6. Table of gene expression measured by qPCR and expressed as fold changes between mutants in biofilm and planktonic cultures. Data were collected in duplicate, and error represents the standard error of the mean

	**Planktonic**	**Biofilm**
Δngo-Lig E	ND	ND
ngo-Lig E-His	1.022 (± 0.061)	1.081 (± 0.008)
opaB-ngo-Lig E	88.62 (± 1.282)	120.423 (± 22.244)

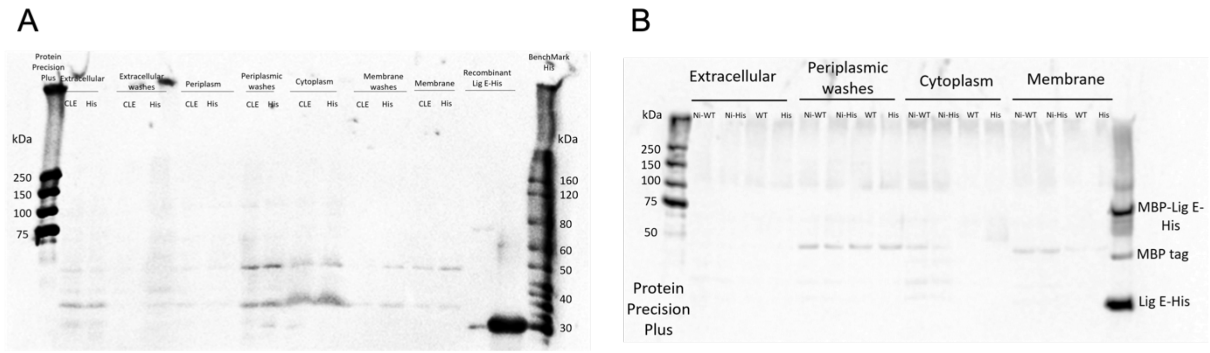

Supplement 7. Western blots of different cellular fractions of N. gonorrhoeae MS11 using an anti-His antibody. Ladders are indicated in kDa. Sizes of the recombinant protein controls are 32kDa for Lig E-His, 44 kDa for the MBP tag and 76 kDa for MBP-Lig E-His. A) Comparison of the different fractions from the His-tagged constitutively upregulated Ngo-Lig E (opaB-Ngo-Lig E; CLE) and the His-tagged Ngo-lig E (ngo-Lig E-His; His) mutants. B) Comparisons of fractions from wild-type N. gonorrhoeae (WT) and the His-tagged Ngo-lig E (ngo-Lig E-His; His) as well as the respective nickel pull downs of each fraction (Ni-WT and Ni-His respectively).

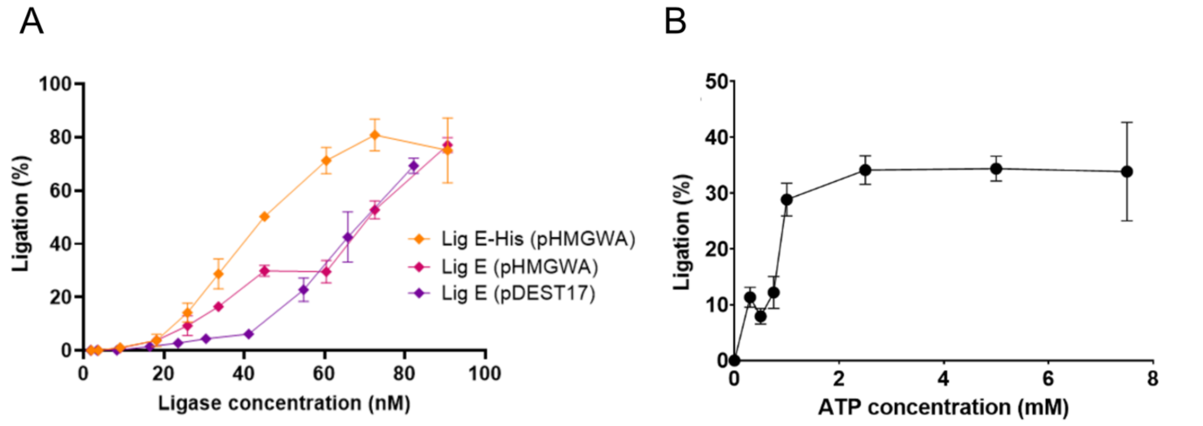

Supplement 8. A) Ligase activity on singly-nicked DNA substrate for various recombinantly-produced Ngo-Lig E constructs; mature Ngo-Lig E purified from N-terminal His-tagged fusion (pDEST17) with the tag removed, Ngo-Lig E purified from N-terminal MBP-fusion (pHMGWA) with the tag removed and Ngo-Lig E purified from N-terminal MBP-fusion (pHMGWA) with the tag removed. B) ATP optimum of Ngo-Lig E. Measurements were made in triplicate, error represented the standard deviation of the mean.

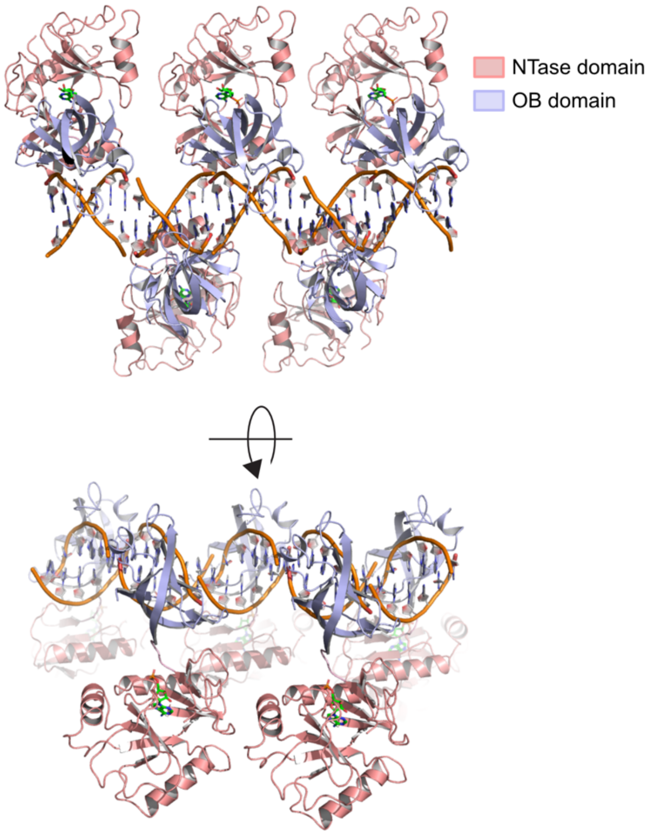

Supplement 9. Symmetry-related molecules in the Ngo-Lig E crystal. Symmetry-related mates within 4 Å are shown generating the continuous DNA filament are shown.

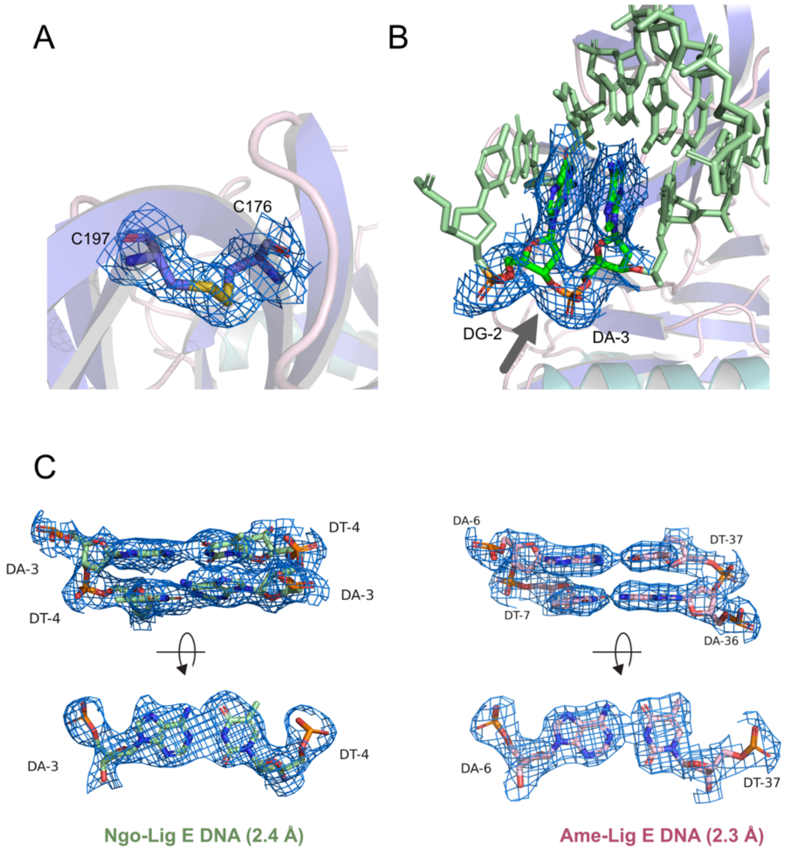

Supplement 10. Observed electron density map (2Fo-Fc) surrounding key features of the Ngo-Lig E structure including: A) the disulphide bond, displayed at the 1.0 □ level. B) The DNA strand that is equivalent to the lick in other DNA ligase-DNA structures, shoing continuous density in the present structure. Map is displayed at the 0.5 □ level. C) Comparison of density surrounding centrally-placed base-pairs in the Ngo-Lig E structure and the equivalent base-pair in the Alteromonas mediterranea (Ame-Lig E) 6GDR.
